# Supplementary material for: Robust 2D layered MXene matrix–boron carbide hybrid films for neutron radiation shielding
Source: Nat Commun. 2023 Oct 31;14:6957. doi: 10.1038/s41467-023-42670-z (PMC10618517; doi:10.1038/s41467-023-42670-z)
Supplement: Supplementary file 1 — Supplementary Information [file 41467_2023_42670_MOESM1_ESM.pdf]

Supplementary Information for

Robust 2D layered MXene matrix – boron carbide hybrid films for neutron radiation shielding

Ju-Hyoung Han[1,6], Shi-Hyun Seok[1,6], Young Ho Jin[1], Jaeeun Park[1], Yunju Lee[2], Haeng Un Yeo[1], Jong-Ho Back[3], Yeoseon Sim[1], Yujin Chae[1], Jaewon Wang[1], Geum-Yoon Oh[4], Wonjoo Lee[3], Sung Hyun Park[4], In-Cheol Bang[2], Ji Hyun Kim[2], Soon-Yong Kwon[1,5]*

[1]*Department of Materials Science and Engineering, Ulsan National Institute of Science and Technology (UNIST), Ulsan 44919, Republic of Korea*

[2]*Department of Nuclear Engineering, Ulsan National Institute of Science and Technology (UNIST), Ulsan 44919, Republic of Korea*

[3]*Center for Advanced Specialty Chemicals, Korea Research Institute of Chemical Technology (KRICT), Ulsan 44412, Republic of Korea*

[4]*Sustainable Technology and Wellness R&D Group, Korea Institute of Industrial Technology (KITECH), Jeju 63243, Republic of Korea*

[5]*Graduate School of Semiconductor Materials and Devices Engineering, Ulsan National Institute of Science and Technology (UNIST), Ulsan 44919, Republic of Korea*

[6]*These authors contributed equally.*

*Correspondence should be addressed. Email to: sykwon@unist.ac.kr

- Supplementary Notes 1-3
- Supplementary Figures 1-23
- Supplementary Tables 1-6
- Supplementary References 1-25

Supplementary Note 1. Synthesis and characterizations of high quality $Ti_3C_2T_x$ MXene

The high crystallinity of the Ti_3AlC_2 MAX phase and exfoliated $Ti_3C_2T_x$ MXene was confirmed via XRD analysis (Supplementary Figure 1a). No other crystalline impurities, such as Ti_2AlC, Al_2O_3, and TiC, which could greatly increase the defects of the subsequent MXene structure,[1] was observed in the XRD pattern, indicating that the Ti_3AlC_2 MAX phase was ultrapure (black line in Supplementary Figure 1a). After etching and delamination, the (10*n*) peaks disappeared and the (002) peak shifted to a lower angle from $2\theta \approx 9.48°$ to 7.04°, which corresponds to the shift in *d*-spacing from 9.32 Å to 12.55 Å, due to the removal of Al in Ti_3AlC_2 and exfoliation of MXene, similar to that in the literature.[2] SEM image of the delaminated $Ti_3C_2T_x$ (Supplementary Figure 1b) and corresponding statistical analysis (Supplementary Figure 1c) confirmed that the lateral size of the delaminated $Ti_3C_2T_x$ flakes was in the range of several micrometers. TEM image showed that the $Ti_3C_2T_x$ flake had relatively clean edge and surface without oxidized particles. SAED pattern revealed that the $Ti_3C_2T_x$ flake was a single crystal with a hexagonal atomic arrangement, in agreement with a previous report (Supplementary Figure 1d).[3] The height profile obtained by AFM analysis revealed a successful delamination of $Ti_3C_2T_x$ into a single layer, which had a thickness of ≈1.7 nm (Supplementary Figure 1e). The Raman spectrum exhibited prominent peaks that can be assigned to out-of-plane vibration modes (A_{1g}) and in-plane vibration modes (E_{1g}), which is consistent with a previous study on the vibrational properties of MXenes (Supplementary Figure 1f).[4] Notably, there were no signs of the D and G bands representing disordered and graphitic C atoms originated from surface defects of $Ti_3C_2T_x$, which is in a good agreement with the TEM observation.[5] The chemical composition of $Ti_3C_2T_x$ was investigated using XPS (Supplementary Figure 2). The XPS spectra of the Ti 2*p* region showed that the peaks at 454.7 eV ($2p_{3/2}$) and 461.0 eV ($2p_{1/2}$) were assigned to the Ti-C bonds, and the peaks at 455.7 eV ($2p_{3/2}$) and 461.8 eV ($2p_{1/2}$), 457.0 eV ($2p_{3/2}$) and 463.0 eV ($2p_{1/2}$) were consistent with Ti^{2+} and Ti^{3+} states, respectively (Supplementary Figure 2a).[6,7] The XPS spectra of O 1*s*, F 1*s*, and Cl 2*p* revealed the presence of surface terminations (i.e., -OH, -F, -O, and -Cl) of $Ti_3C_2T_x$ that were produced during the exfoliation process (Supplementary Figure 2c-e). The XPS results are summarized in Supplementary Table 1. According to the above characterizations, the as-prepared $Ti_3C_2T_x$ MXene flakes exhibited a high quality. The freestanding $Ti_3C_2T_x$ films were fabricated via vacuum-assisted filtration of the delaminated MXene solutions (Supplementary Figure 1h). They exhibited excellent flexibility without any fracture (Supplementary Figure 1i) and showed great foldability without deformation because of the desirable mechanical properties of MXene flakes with large lateral sizes and a structured hydrogen-bonding network formulated by the interfacial water.[8]

Supplementary Note 2. Preparation of stable dispersion of the n-B_4C powder

A stable dispersion of the B_4C powder with particle sizes of few hundreds of nanometers was prepared to fabricate a hybrid film with exfoliated MXene flakes. The as-received ^{10}B rich-B_4C (AR-B_4C) that was commercially available (3M Technical Ceramics, B_4C powder, ^{10}B >95%) powder formed a heterogeneous dispersion in water such that the upper layer had a thin and the lower layer had a thick brownish color owing to the gravitational settling effect (Supplementary Figure 3a). When the $Ti_3C_2T_x$ aqueous solution was mixed with AR-B_4C, this hybrid solution was also settled down even if the MXene dispersion was stable (Supplementary Figure 3b). SEM and EDS mapping images of the sediment showed aggregation of $Ti_3C_2T_x$ flakes (marked by yellow arrows) on large B_4C particles (Supplementary Figure 3c). In addition, it was demonstrated that vacuum-filtrated films of the hybrid solution (AR-$B_4C/Ti_3C_2T_x$) could not maintain structural integrity owing to the large number of cracks (Supplementary Figure 4a). Compared with the cross-sectional SEM image of the pristine $Ti_3C_2T_x$ freestanding film (inset in Supplementary Figure 4b), AR-$B_4C/Ti_3C_2T_x$ lost its densely packed and aligned structure because of the presence of macroscopic pores (marked by yellow ellipses), which probably resulted from the large particle size of AR-B_4C (Supplementary Figure 4b). The XRD pattern of AR-$B_4C/Ti_3C_2T_x$ showed that the characteristic (002) peak of $Ti_3C_2T_x$ was broadened and shifted to a lower angle (i.e., to a larger *d*-spacing), corresponding to the incorporation of AR-B_4C particles and the decline of $Ti_3C_2T_x$ flake alignment (Supplementary Figure 4c).

To fabricate a robust and scalable film toward practical applications, a homogenous dispersion of B_4C and its hybrid dispersion with $Ti_3C_2T_x$ had to be achieved before the fabrication of the film to reduce the large particles of B_4C, which induce the aggregation of $Ti_3C_2T_x$ flakes and pores, eventually resulting in a fracture of the hybrid film. DLS analysis revealed that the particles of AR-B_4C had a broad and bimodal distribution composed of relatively small (z-average of ≈600 nm) and large (z-average of ≈5.5 μm) regions (black line in Supplementary Figure 5). After 2 h of bath sonication and removal of the sediment containing large particles through centrifugation, AR-B_4C particles were reduced to smaller ones (hereafter, n-B_4C), and the distribution of n-B_4C exhibited a sharp peak intensity with a z-average of ≈150 nm (red line in Supplementary Figure 5). SEM observations of AR-B_4C indicated that the particles were between several hundred nanometers and a few micrometers in size, which is in good agreement with the DLS analysis (Supplementary Figure 6a). Smaller particles showing markedly different contrasts were observed in the SEM image of n-B_4C, showing a similar tendency for the DLS-derived size distribution, as reported in a previous study (Supplementary Figure 6b).[9] It is well known that B_4C with small particle sizes can be easily oxidized in water or air owing to its large surface area.[10,11] An EDS comparison between AR-B_4C and n-B_4C revealed that n-B_4C had more oxygen species than AR-B_4C, which may be the cause of the contrast in the SEM image (Supplementary Figure 6c). The compositions of

AR-B_4C and n-B_4C had B, C, and O contents with atomic ratios of 4:0.89:0.005 and 4:1.096:0.046, respectively, as calculated from the EDS results (Supplementary Table 2). The TEM image showed that the surface of n-B_4C was covered by an amorphous layer (marked by yellow arrow), which was assumed to be boron oxide (Supplementary Figure 7). FT-IR spectra further proved that n-B_4C had a more oxidized moiety with a broad O–H band at ≈ 3,400 cm^{-1} and a B–O band at ≈ 1,400 cm^{-1}, suggesting the presence of adsorbed water or hydroxyl groups on the surface of B_4C (Supplementary Figure 8a).[12] Furthermore, the strong vibrations at ≈ 1,117 cm^{-1} and peak at ≈ 1,619 cm^{-1}, attributed to the typical vibration bands of boron carbide, were slightly shifted to lower wavenumbers (Supplementary Figure 8b). This can be interpreted as the vibration of the typical characteristic peak of B_4C being weakened by more oxide bonding on the surface of B_4C, which is in accordance with the EDS and TEM analyses. Nevertheless, the XRD pattern showed that n-B_4C contained no trace of B_2O_3, whereas the ball-milling process, which is a conventional method to reduce particle size, induced impurities, including Fe and B_2O_3, because of the severe contamination from the systems and oxidation occurring during the process (Supplementary Figures 9a and b). These results indicate that the surface chemical state of n-B_4C was modified to enhance electrostatic dispersibility, maintaining structural integrity and high crystallinity, as discussed later. We also compared the dispersion and yield of the n-B_4C prepared with and without sonication. It was found that the sonication process promoted the breakage of the B_4C particles into smaller sizes, increasing the concentration and yields without compromising the crystal structure and the purity of the sample (Supplementary Table 3).

Supplementary Note 3. Preparation of electrostatically stable MXene / B_4C aqueous dispersion

Generally, a stable colloidal dispersion is characterized by zeta (ζ) potentials other than +30 mV or -30 mV.[13] This implies that the dispersion maintains its form without any aggregation or flocculation owing to electrostatic repulsion between the charged particles. With the help of the surface boron hydroxide providing electronegativity, n-B_4C particles exhibited high ζ potential value at -52.2 mV (Supplementary Figure 10a).[14] Even though AR-B_4C had a negative ζ potential value at -40.1 mV, which is sufficient to form a stable colloid, the large particles could not resist the gravitational force, as discussed above. To investigate the effect of the particle size on the interaction between $Ti_3C_2T_x$ and B_4C, large B_4C (L-B_4C) particles were isolated via centrifugation and decantation. The ζ potential of L-B_4C exhibited a broader and higher distribution compared with those of AR-B_4C and n-B_4C. Despite the negative average value of L-B_4C, a positive region was clearly observed in the ζ potential, which suggests the presence of an electrostatic attraction force between L-B_4C and MXene. This force results in the aggregation of MXene flakes on the surfaces of larger B_4C particles, as shown in Supplementary Figure 3. Eventually, with the help of repulsive forces between two different materials, a stable hybrid dispersion of n-B_4C and $Ti_3C_2T_x$ with a ζ potential of -38.2 mV was achieved (Supplementary Figure 10b). All the hybrid solutions, with weight fractions of n-B_4C ranging from 0 to 100 wt%, maintained stability without any aggregation or sedimentation even after 7 days. The importance of the homogeneity of the hybrid solution was further confirmed using vacuum-filtrated films. The SEM image of AR-B_4C/$Ti_3C_2T_x$ showed that it had a rough surface caused by the extruded large B_4C particles (Supplementary Figure 11a). Moreover, the EDS mapping results showed that AR-B_4C/$Ti_3C_2T_x$ had a non-uniform distribution of boron, which could deteriorate the effectiveness of neutron radiation shielding.[15] However, a more even surface as well as homogenous distribution of boron was observed on the vacuum-filtrated film of the hybrid dispersion of n-B_4C and $Ti_3C_2T_x$ (n-B_4C/$Ti_3C_2T_x$) (Supplementary Figure 11b).

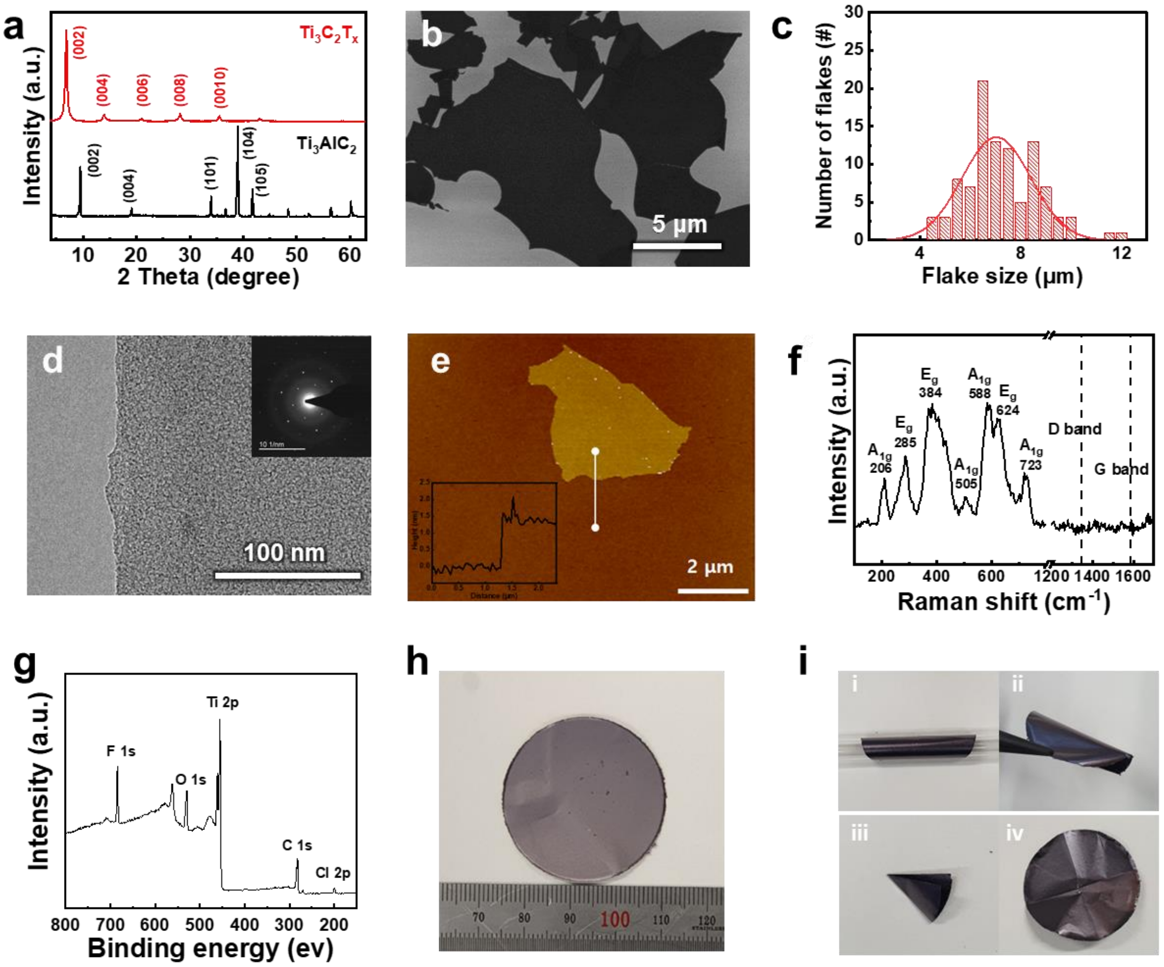

Supplementary Figure 1. Characterizations of high quality $Ti_3C_2T_x$ MXene. (a) XRD patterns of Ti_3AlC_2 MAX phase (black line) and $Ti_3C_2T_x$ (red line). (b) SEM image of $Ti_3C_2T_x$ flakes drop-casted on the SiO_2 wafer. (c) Statistical analysis of size distribution of $Ti_3C_2T_x$ MXene flakes. (d) TEM image and SAED pattern (inset) of the single-layer $Ti_3C_2T_x$ flake. (e) AFM image of $Ti_3C_2T_x$ flake. Inset of figure indicated that height of $Ti_3C_2T_x$ flake ≈1.7 nm, respectively. (f) Raman spectrum of $Ti_3C_2T_x$. Vertical dashed line indicates the position of D band and G band. (g) XPS spectrum of $Ti_3C_2T_x$. (h) Photograph of vacuum-filtrated freestanding $Ti_3C_2T_x$ film which has a diameter ≈40 mm. (i) Photograph of $Ti_3C_2T_x$ film which showing good flexibility of the $Ti_3C_2T_x$ film. Rolled on glass rod (i-i), released (i-ii), Folded (i-iii), and unfolded (i-iv).

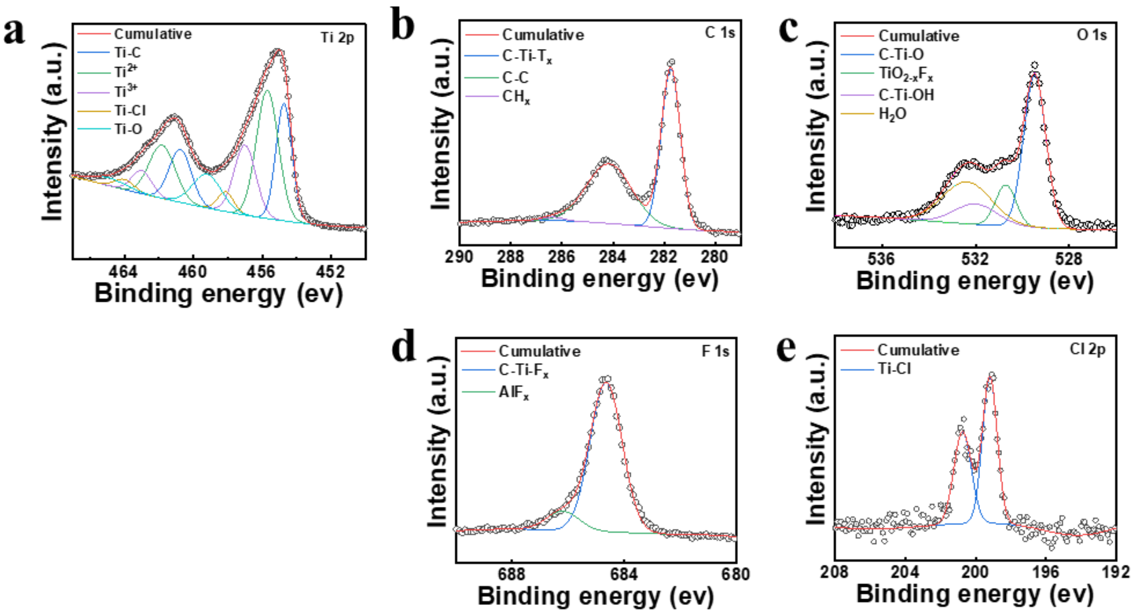

Supplementary Figure 2. XPS analysis of the $Ti_3C_2T_x$. Spectra of (a) Ti 2*p*, (b) C 1*s*, (c) O 1*s*, (d) F 1*s*, and (e) Cl 2*p*.

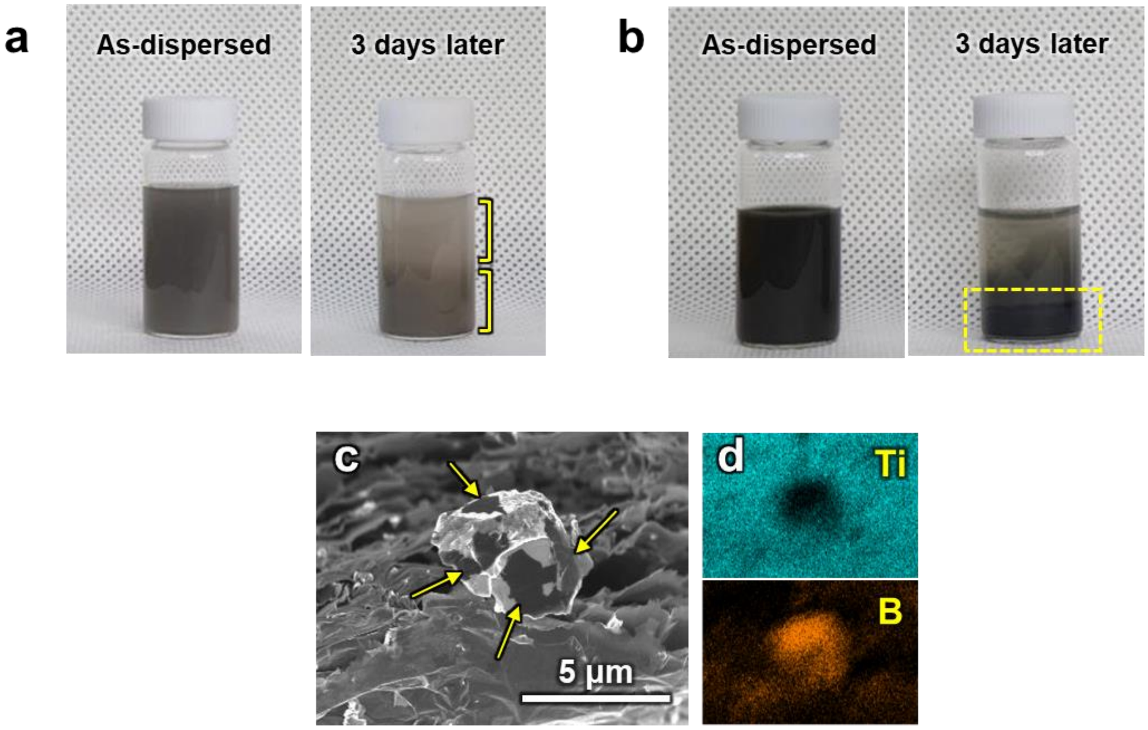

Supplementary Figure 3. Aggregation behavior of AR-$B_4C/Ti_3C_2T_x$ (a) Photographs of AR-B_4C as dispersed and 3 days later in the aqueous media. Heterogeneous dispersion state was put in a yellow bracket. (b) Photographs of hybrid dispersion of AR-B_4C and $Ti_3C_2T_x$ as dispersed and 3 days later. Dashed yellow box represents aggregated and sedimented slurry. (c) SEM image of the large B_4C particle with attached MXene flakes marked with yellow arrow. (d) Corresponding EDS mapping results of Ti and B.

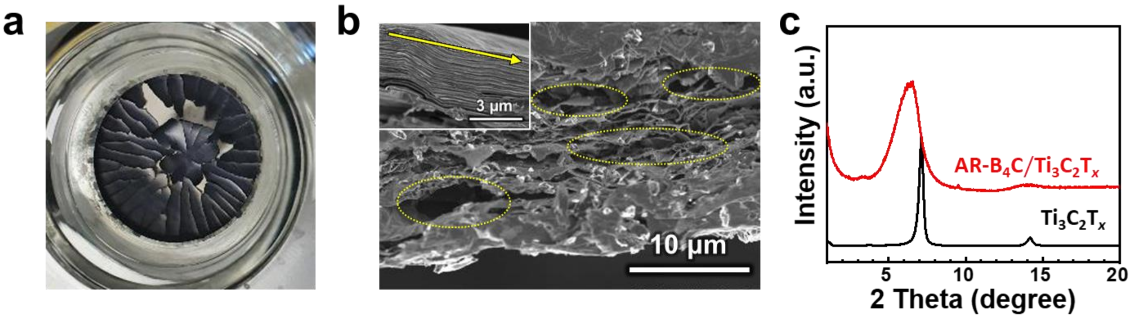

Supplementary Figure 4. Unstable structure of a fabricated AR-$B_4C/Ti_3C_2T_x$ film. (a) Photograph of AR-$B_4C/Ti_3C_2T_x$. Several cracks can be seen. (b) Cross sectional SEM image of vacuum-filtrated films of AR-$B_4C/Ti_3C_2T_x$ hybrid solution (AR-$B_4C/Ti_3C_2T_x$) and pristine $Ti_3C_2T_x$ dispersion (inset). Characteristic aligned structure of was confirmed in the $Ti_3C_2T_x$ film. For the sake of clarity, the aligned direction and micropores are highlighted by a yellow arrow and ellipses, respectively. (c) XRD pattern of $Ti_3C_2T_x$ (black) and AR-$B_4C/Ti_3C_2T_x$ (red). The peak was broadened and shifted to lower angle, implying detriment of aligned structure and increased *d*-spacing by incorporating AR-B_4C particles.

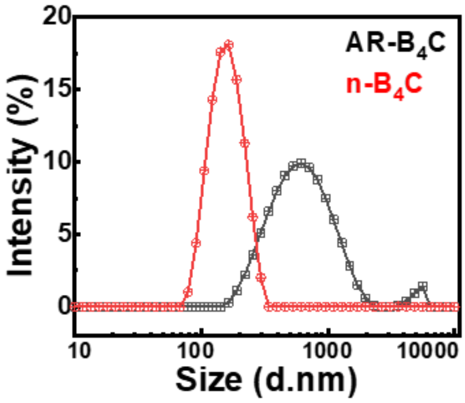

Supplementary Figure 5. Size distribution obtained by the DLS analysis of AR-B_4C (black dotted line) and n-B_4C (red dotted line).

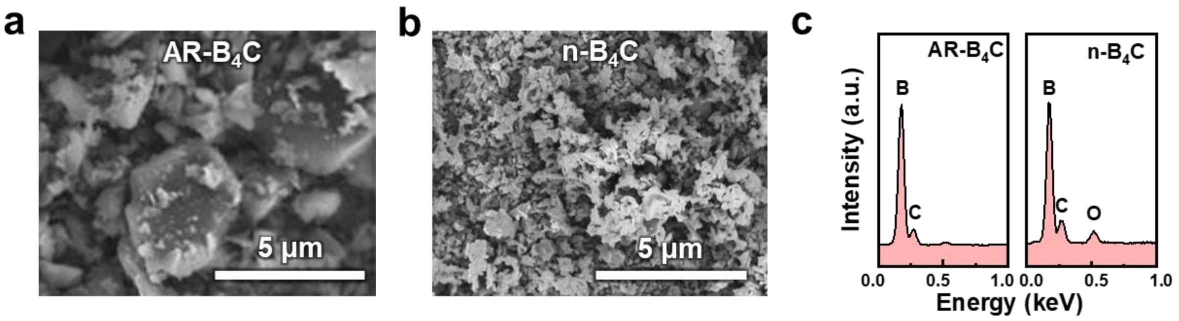

Supplementary Figure 6. Morphologies of AR- and n-B_4C. Representative SEM images of (a) AR-B_4C and (b) n-B_4C. (c) EDS spectra of AR-B_4C and n-B_4C. Each element of B_4C was labelled on the spectrum.

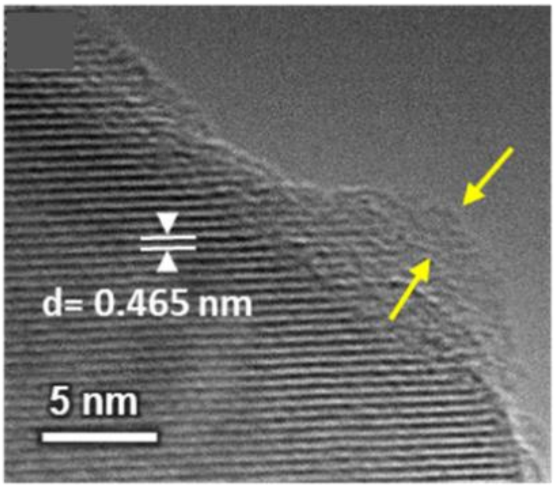

Supplementary Figure 7. TEM image of n-B_4C. The surface of the n-B_4C particle is marked by two yellow arrows.

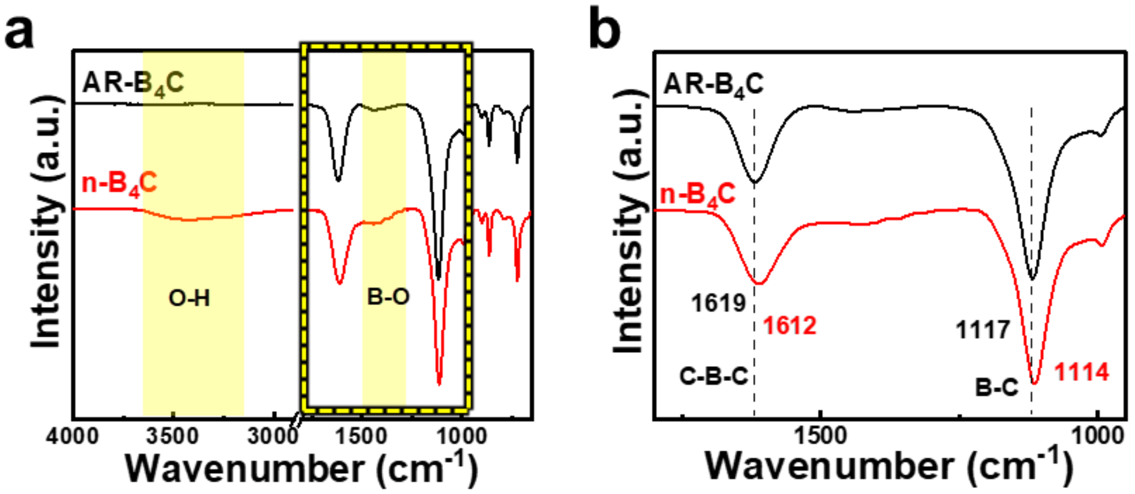

Supplementary Figure 8. FT-IR study of AR- and n-B_4C. (a) FT-IR spectra of AR-B_4C and n-B_4C. The areas highlighted by the yellow rectangle represent O–H and B–O vibrations. (b) Enlarged FT-IR spectrum highlighted with dashed yellow box from (a). Vertical dashed line represents characteristic vibration mode of B_4C.

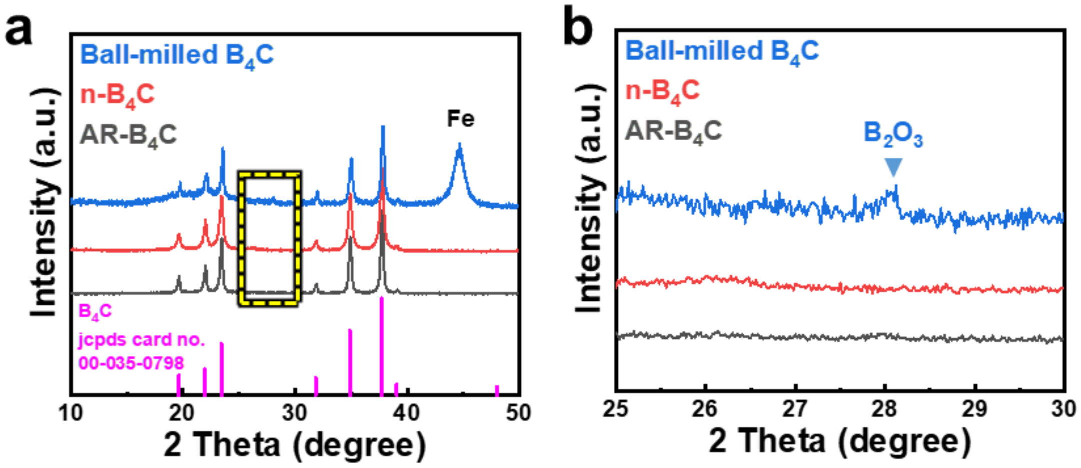

Supplementary Figure 9. XRD patterns of AR- and n-B_4C compared with ball-milled B_4C. (a) XRD pattern of AR-B_4C (black), n-B_4C (red), ball-milled B_4C (blue), and the pattern of rhombohedral B_4C corresponding to JCPDS card no. 00-035-0798 (magenta). Due to the strong hardness of the B_4C, high energy ball-mill was conducted, which caused contamination of the B_4C powder. (b) Enlarged XRD pattern of (a) which is highlighted by yellow rectangular box. Besides contamination from ball and container, accelerated oxidation occurred due to heat and energy during the ball-milling process.

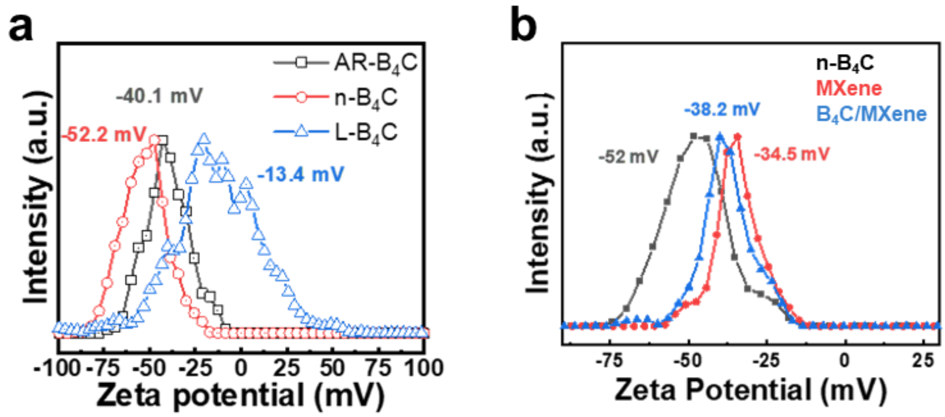

Supplementary Figure 10. Zeta potential analysis. (a) AR-B_4C (black), n-B_4C (red), and L-B_4C (blue). (b) n-B_4C (black), $Ti_3C_2T_x$ MXene (red), and MB hybrid (blue).

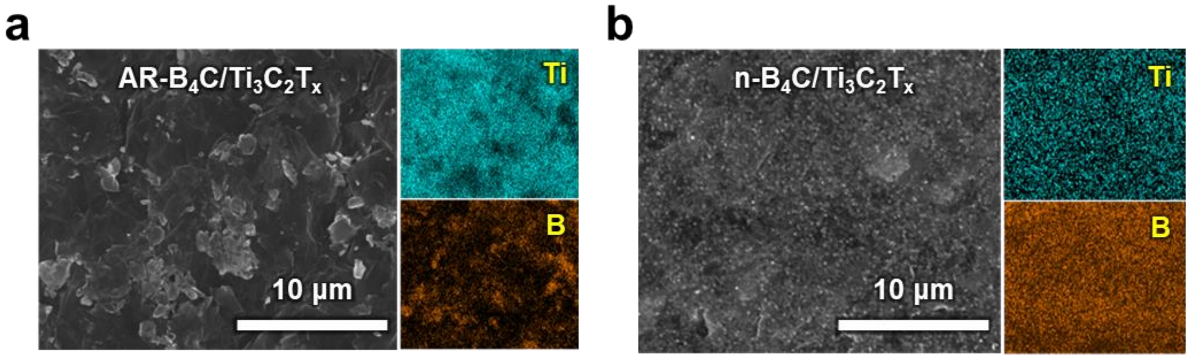

Supplementary Figure 11. Structural characterization of $B_4C/Ti_3C_2T_x$ hybrid vacuum-filtrated films. (a) SEM image of AR-$B_4C/Ti_3C_2T_x$ vacuum filtrated film. Extruded surface morphology can be found due to large particle size of AR-B_4C. Corresponding EDS mapping analysis indicated non-uniform distribution of Boron element. (b) SEM image and corresponding EDS mapping results of Ti and B of n-$B_4C/Ti_3C_2T_x$.

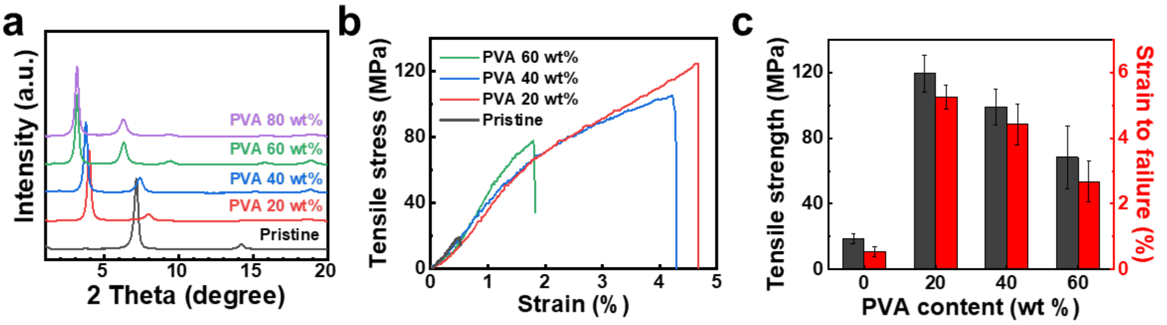

Supplementary Figure 12. XRD studies of $Ti_3C_2T_x$/PVA composite films with different weight fractions. (a) XRD patterns of $Ti_3C_2T_x$/PVA composite films with different PVA weight fractions. (b) Stress-strain curves and (c) tensile strengths and strains to failure of the composite films with different PVA contents. The average and standard deviation in (c) are represented as bar graphs ± error bars, obtained from three different samples in each PVA content. The error bars are 3.2, 11.3, 10.9, and 19.2 MPa (from left to right).

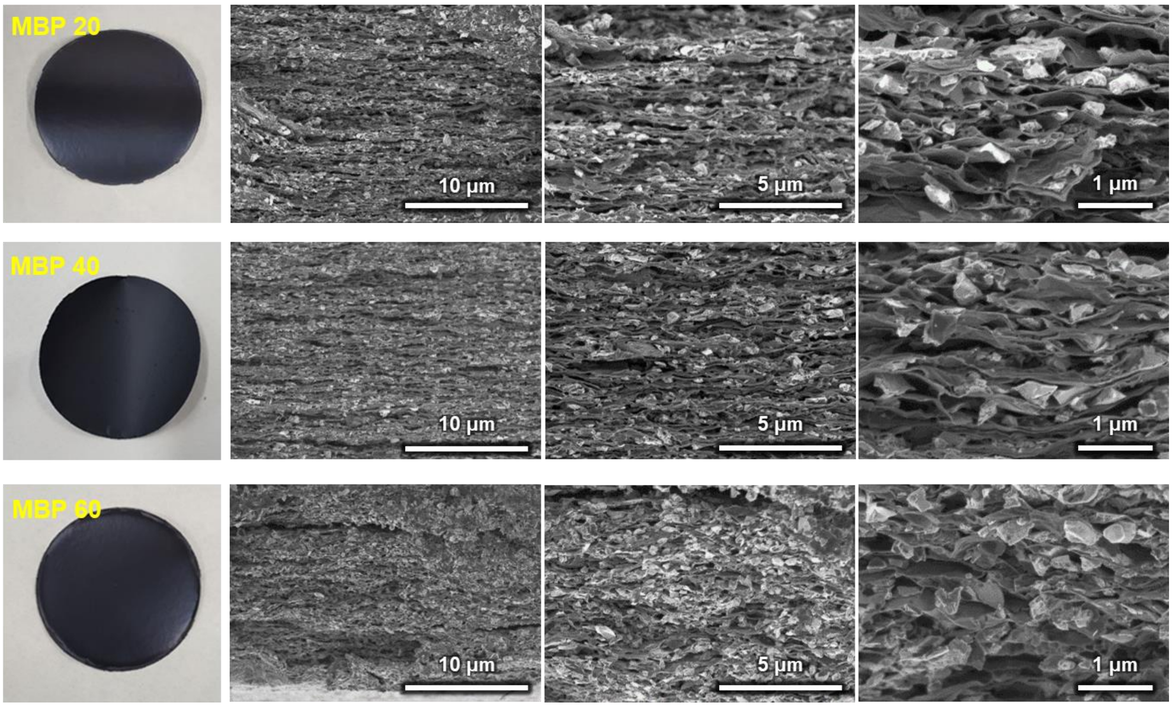

Supplementary Figure 13. **Photographs of the freestanding MBP hybrid films with the different B_4C fraction of 20–60 wt% and cross-sectional SEM images showing the highly ordered in-plane orientation with the incorporated B_4C between the $Ti_3C_2T_x$ MXene flakes and PVA layers.**

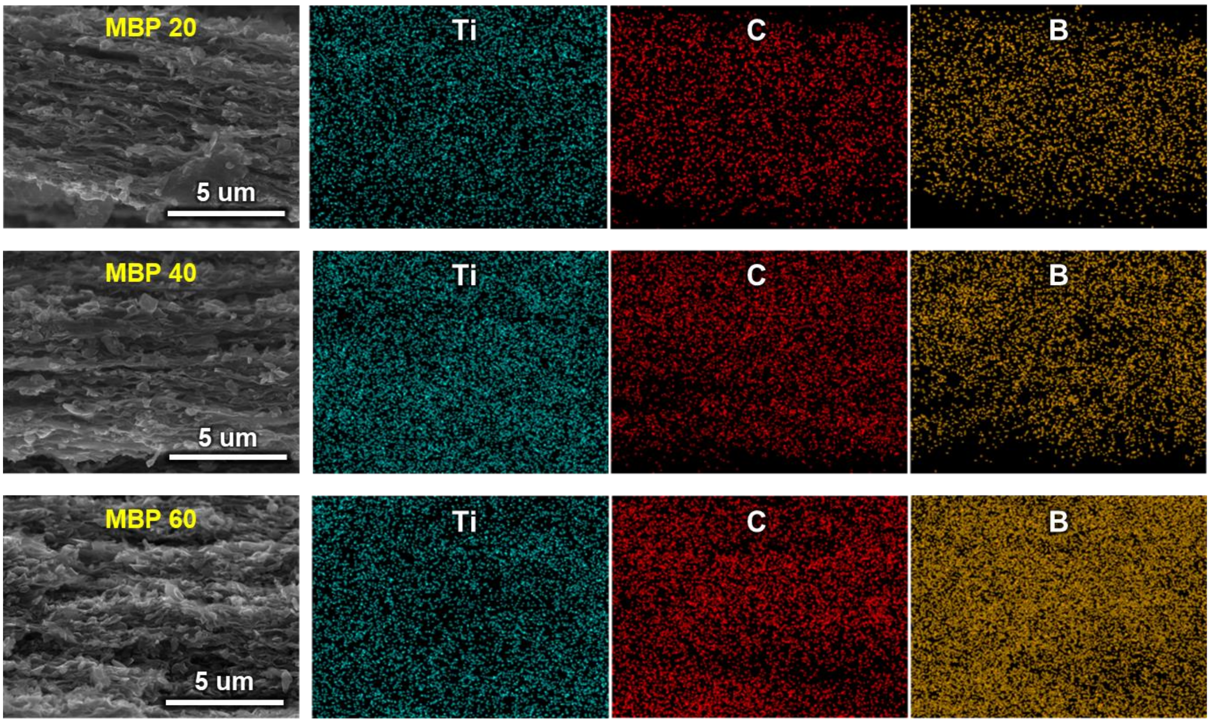

Supplementary Figure 14. **Cross-sectional EDS mapping images of MBP hybrid films with the different B_4C fraction of 20–60 wt% showing the uniform distribution of boron in the films.**

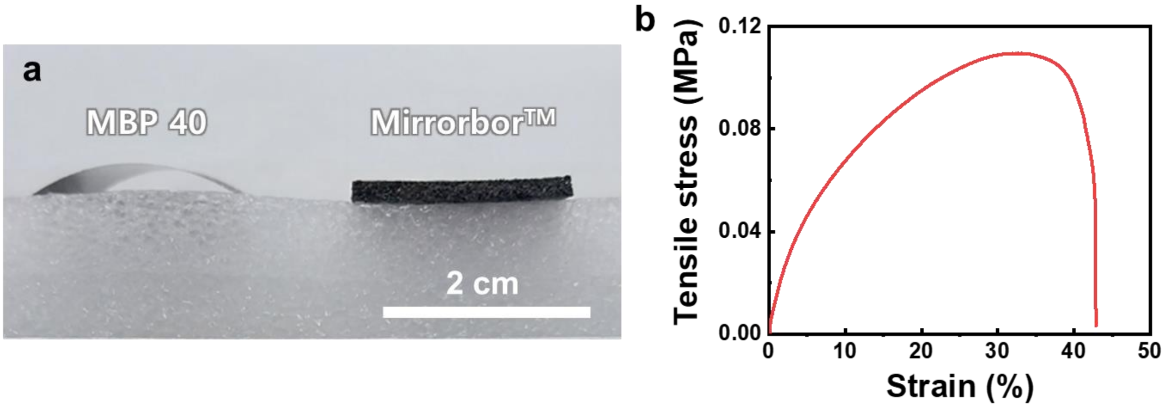

Supplementary Figure 15. Comparison with a commercially available neutron shielding material. (a) Photograph of MBP 40 and Mirrorbor^{TM}. (b) Stress-strain curves of Mirrorbor^{TM}.

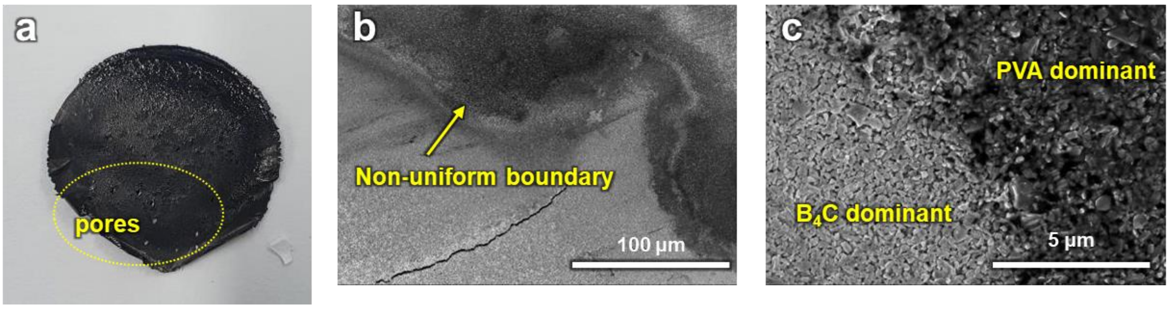

Supplementary Figure 16. **Importance of $Ti_3C_2T_x$ as a platelet for hybrid composites.** (a) A photograph of vacuum-filtrated n-B_4C/PVA composite film without $Ti_3C_2T_x$ MXene flakes. (b and c) SEM images showing the separation of separation of B_4C particles and PVA matrix with the nonuniformity of film.

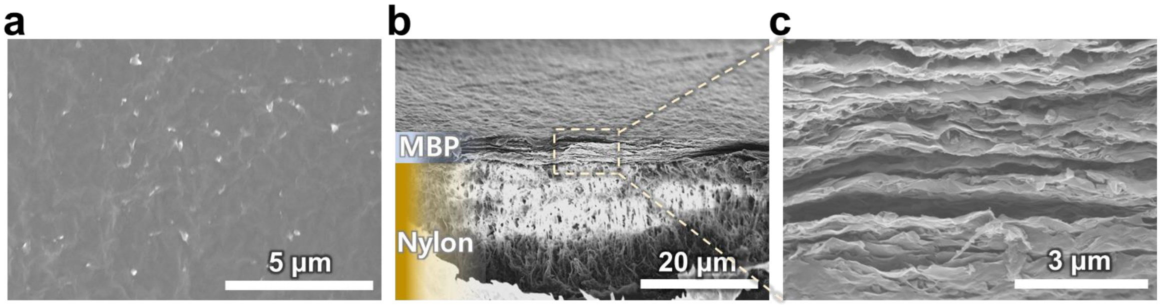

Supplementary Figure 17. **Morphologies of the painted MBP hybrid film on a nylon fabric membrane.** SEM images of (a) surface and (b and c) cross-section of the blade-coated MBP hybrid film on a nylon membrane.

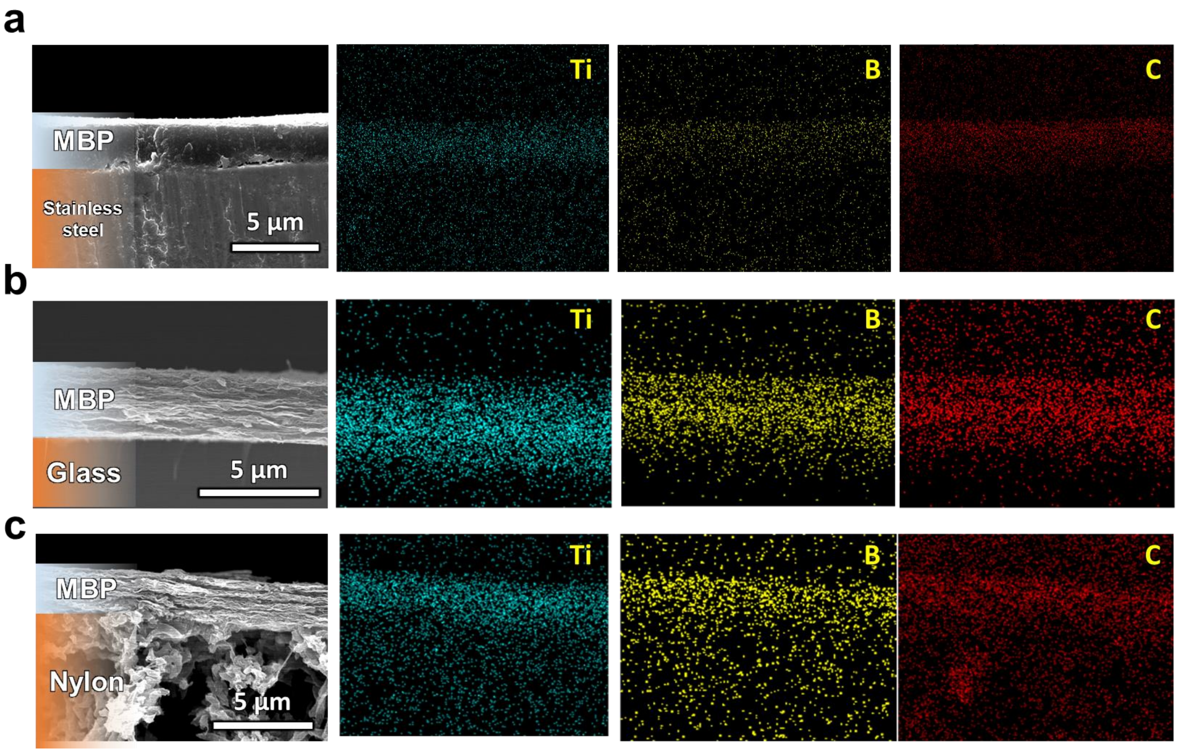

Supplementary Figure 18. **Cross-sectional EDS mapping images of painted MBP hybrid films on a various substrate.** Cross-sectional images of blade-coated MBP hybrid films on (a) stainless-steel, (b) glass, and (c) nylon substrates with a B_4C weight fraction of 40 wt.% showing the uniform distribution of boron in the film.

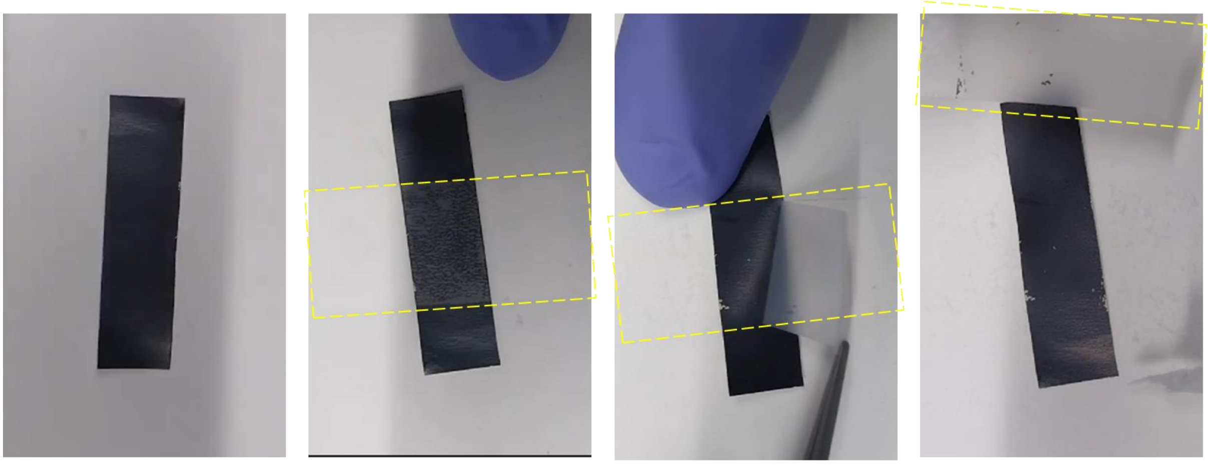

Supplementary Figure 19. **Stripping test on the MBP hybrid film painted on a nylon membrane.**

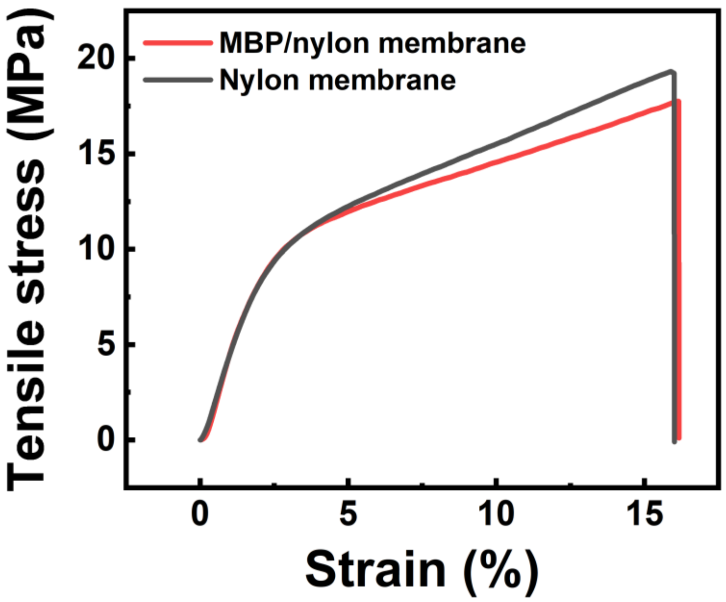

Supplementary Figure 20. **Stress-strain curves of painted MBP hybrid film with a B_4C weight fraction of 40 wt.% on a nylon substrate.**

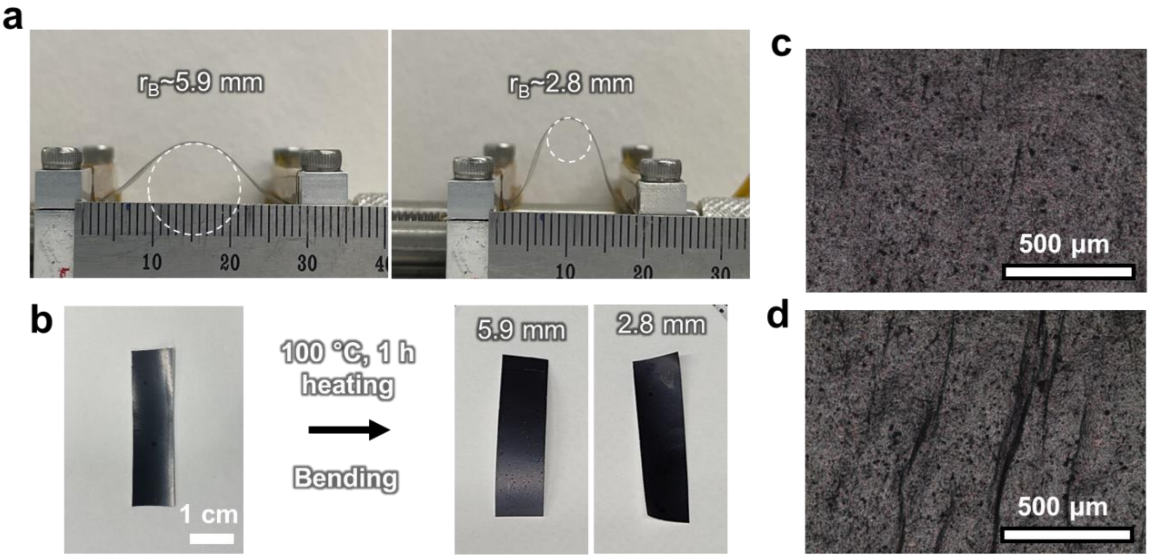

Supplementary Figure 21. **Bent MBP hybrid film/nylon membrane samples.** (a) Bending tests with different bending radii of 5.9 and 2.8 mm. (b) MBP hybrid film/nylon membrane samples before and after repeated bending tests showing that the samples have no noticeable cracks in the area of 1 × 4.5 cm^2. OM images of the (c) released and (d) bent MBP hybrid film/nylon membrane surfaces.

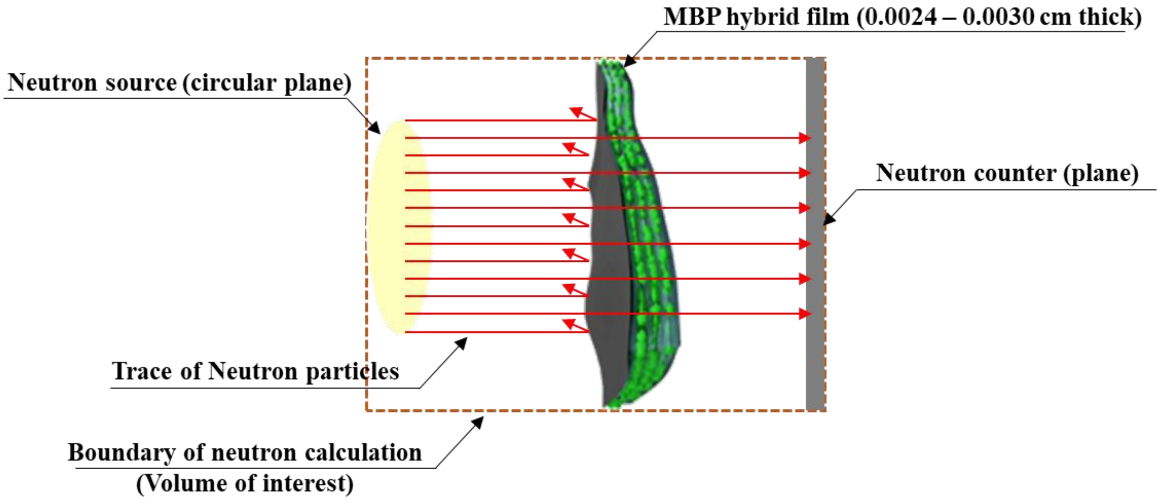

Supplementary Figure 22. Schematic of MCNP simulation on MBP hybrid films.

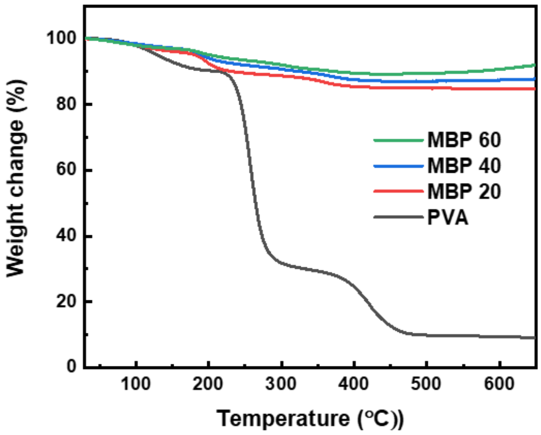

Supplementary Figure 23. **TGA analysis of MBP hybrid films.** TGA curves of MBP hybrid films with varying B_4C contents and a PVA film.

Supplementary Table 1. **Summary of XPS analysis of $Ti_3C_2T_x$.**

Region	BE (eV)	FWHM (eV)	Fraction (%)	Assigned to
Ti $2p_{3/2}$ ($2p_{1/2}$)	454.7 (461.0)	1.1 (1.5)	27.8	Ti-C
	455.7 (461.8)	1.5 (1.8)	38.2	Ti^{2+}
	457.0 (463.0)	1.5 (1.4)	17.9	Ti^{3+}
	458.1 (464.0)	1.1 (1.3)	4.63	Ti-Cl
	459.2 (465.0)	2.0 (2.5)	11.5	Ti-O
C 1*s*	281.7	0.9	52.3	C-Ti-T_x
	284.2	2.1	46.5	C-C
	286.3	1.0	1.1	CH_x
O 1*s*	529.5	1.1	47.3	C-Ti-O
	530.7	1.0	11.8	$TiO_{2-x}F_x$
	532.0	2.1	12.1	C-Ti-OH
	532.4	2.5	28.8	H_2O
F 1*s*	684.6	1.3	88.5	C-Ti-F_x
	686.2	14	11.5	AlF_x
Cl $2p_{3/2}$ ($2p_{1/2}$)	199.2 (200.7)	1.0 (1.2)	100	Ti-Cl

Supplementary Table 2. Calculated atomic ratio from the EDS results corresponding to Supplementary Figure 6.

Element	AR-B_4C	n-B_4C
B at%	81.6	77.7
C at%	18.3	21.3
O at%	0.1	0.9

Supplementary Table 3. Yield comparison of n-B_4C prepared with and without sonication. The yields were measured by vacuum filtration of a 40 mL supernatant after centrifugation.

	**w/o sonication**	**with sonication**
Used AR-B_4C	1 g	1 g
Yield	0.054 g	0.162 g

Supplementary Table 4. Calculated elemental weight fractions of MBP hybrid films.

Samples		^{10}B (wt%)	^{11}B (wt%)	H (wt%)	C (wt%)	O (wt%)	Ti (wt%)
E-MBP	**MBP 20**	15.18	0.47	1.40	24.11	7.43	51.40
	**MBP 40**	30.37	0.94	1.40	25.59	7.43	34.27
	**MBP 60**	45.55	1.40	1.40	27.07	7.43	17.13
N-MBP	**MBP 20**	3.08	12.58	1.40	24.11	7.43	51.40
	**MBP 40**	6.15	25.15	1.40	25.59	7.43	34.27
	**MBP 60**	9.23	37.73	1.40	27.07	7.43	17.13

Supplementary Table 5. **Neutron shielding performance of various shielding materials.**

	Sample	Thickness (cm)	Absorption capacity (%)	Macroscopic cross-section (cm^{-1})	Half-value layer (cm)	Refs.
Commercial plates	Borated stainless steel	1.587	98.5	2.65	2.6×10^{-1}	[16]
	Boral™ (hot-pressed B_4C/Al)	0.1	89.7	22.7	3.1×10^{-2}	
	Metamic™ (B_4C/Al)	0.158	93.1	16.9	4.1×10^{-2}	
Aluminum matrix	TiB_2-Al	1	99.9	7.58	9.1×10^{-2}	[17]
	Cold sprayed B_4C/Al	0.5	55	1.60	4.3×10^{-1}	[18]
	Hot-rolled B_4C (29 wt%)/6061Al alloy	0.158	95.6	19.8	3.5×10^{-2}	[19]
Polymer matrix	30 wt% B_4C/ polyimide	0.08	76	17.8	3.9×10^{-2}	[9]
	Polyethylene/hexagonal boron nitride	0.09	95.8	34	2.0×10^{-2}	[20]
	55 wt% B_4C/ natural rubber latex	0.1	83.5	18	3.9×10^{-2}	[21]
Boron-containing glass	PbO-Sb_2O_3-B_2O_3-CuO	0.34	3.8	0.113	6.1	[22]
	Cr_2O_3/LiF-SrO-B_2O_3	3	27.5	0.107	6.5	[23]
	NiO/B_2O_3-$BaCO_3$-Li_2O_3	1	12.9	0.138	5.0	[24]
Carborane	Carborane/copolyester fibers	0.002	3.4	17.04	4.1×10^{-2}	[25]
**MXene matrix**	**60 wt% ^{10}B enriched-B_4C/$Ti_3C_2T_x$/PVA (filtrated)**	**0.004**	**39.8**	**127.6**	**5.4×10^{-3}**	**This study**
	**40 wt% ^{10}B enriched-B_4C/$Ti_3C_2T_x$/PVA (painted)**	**0.014**	**72.8**	**94.3**	**7.4×10^{-3}**	
	**60 wt% natural B_4C /$Ti_3C_2T_x$/PVA (simulated)**	**0.003**	**11.3**	**39.7**	**1.7×10^{-2}**	
	**40 wt% natural B_4C /$Ti_3C_2T_x$/PVA (simulated)**	**0.003**	**6.9**	**27.7**	**2.5×10^{-2}**	

Supplementary Table 6. Mass densities of MBP hybrid films.

Category	MBP 20	MBP 40	MBP 60
Thickness (μm)	36 ± 2.3	39 ± 2.0	36 ± 2.7
Volume (cm^3)	0.014	0.016	0.014
Weight (g)	0.029	0.030	0.029
Density (g cm^{-3})	2.00	1.95	2.02

Supplementary References

1 Scheibe, B., Kupka, V., Peplinska, B., Jarek, M. & Tadyszak, K. The Influence of Oxygen Concentration during MAX Phases (Ti_3AlC_2) Preparation on the alpha-Al_2O_3 Microparticles Content and Specific Surface Area of Multilayered MXenes ($Ti_3C_2T_x$). *Materials (Basel)* **12** (2019).

2 Alhabeb, M. *et al.* Guidelines for Synthesis and Processing of Two-Dimensional Titanium Carbide ($Ti_3C_2T_x$ MXene). *Chem. Mater.* **29**, 7633-7644 (2017).

3 Chen, H. *et al.* Pristine Titanium Carbide MXene Films with Environmentally Stable Conductivity and Superior Mechanical Strength. *Adv. Funct. Mater.* **30** (2019).

4 Sarycheva, A. & Gogotsi, Y. Raman Spectroscopy Analysis of the Structure and Surface Chemistry of $Ti_3C_2T_x$ MXene. *Chem. Mater.* **32**, 3480-3488 (2020).

5 Zhang, C. J. *et al.* Oxidation Stability of Colloidal Two-Dimensional Titanium Carbides (MXenes). *Chem. Mater.* **29**, 4848-4856 (2017).

6 Halim, J. *et al.* X-ray photoelectron spectroscopy of select multi-layered transition metal carbides (MXenes). *Appl. Surf. Sci.* **362**, 406-417 (2016).

7 Benchakar, M. *et al.* One MAX phase, different MXenes: A guideline to understand the crucial role of etching conditions on $Ti_3C_2T_x$ surface chemistry. *Appl. Surf. Sci.* **530**, 147209 (2020).

8 Zheng, S. *et al.* Multitasking MXene Inks Enable High-Performance Printable Microelectrochemical Energy Storage Devices for All-Flexible Self-Powered Integrated Systems. *Adv. Mater.* **33**, 2005449 (2021).

9 Li, X. *et al.* High temperature resistant polyimide/boron carbide composites for neutron radiation shielding. *Compos. B. Eng.* **159**, 355-361 (2019).

10 Lawrence M. Litz, R. A. M. Oxidation of Boron Carbide by Air, Water, and Air-Water Mixtures at Elevated Temperatures. *Reduction of Dicyandiamide* **110**, 921 (1963).

11 Li, Y. Q. & Qiu, T. Oxidation behaviour of boron carbide powder. *Mater. Sci. Eng., A* **444**, 184-191 (2007).

12 Li, X. *et al.* The dispersion of boron carbide powder in aqueous media. *J. Eur. Ceram. Soc.* **33**, 1655-1663 (2013).

13 Joseph, E. & Singhvi, G. in *Nanomaterials for Drug Delivery and Therapy* 91-116 (2019).

14 Mortensen, M. W. *et al.* Preparation and characterization of Boron carbide nanoparticles for use as a novel agent in T cell-guided boron neutron capture therapy. *Appl Radiat Isot* **64**, 315-324 (2006).

15 Soltani, Z., Beigzadeh, A., Ziaie, F. & Asadi, E. Effect of particle size and percentages of Boron carbide on the thermal neutron radiation shielding properties of HDPE/B_4C composite: Experimental and simulation studies. *Radiat. Phys. Chem.* **127**, 182-187

(2016).

16 Choi, J.-S. *et al.* Application of neutron-absorbing structural-amorphous metal (SAM) coatings for spent nuclear fuel (SNF) container to enhance criticality safety controls. *MRS Online Proceedings Library* **985**, 802 (2007).

17 Wang, C. *et al.* *Research on Thermal Neutron Shielding Performance of TiB_2-Al Composite Materials.* https://doi.org/10.26434/chemrxiv.13611725.v1 (2021).

18 Tariq, N. H. *et al.* Cold spray additive manufacturing: A viable strategy to fabricate thick B_4C/Al composite coatings for neutron shielding applications. *Surf. Coat. Technol.* **339**, 224-236 (2018).

19 Lee, D. *et al.* Mechanical and thermal neutron absorbing properties of B_4C/aluminum alloy composites fabricated by stir casting and hot rolling process. *Metals* **11**, 413 (2021).

20 Shang, Y. *et al.* Multilayer polyethylene/hexagonal boron nitride composites showing high neutron shielding efficiency and thermal conductivity. *Compos. Commun.* **19**, 147-153 (2020).

21 Liao, Y.-C., Xu, D.-G. & Zhang, P.-C. B_4C/NRL flexible films for thermal neutron shielding. *Nucl. Sci. Tech.* **29**, 17 (2018).

22 Mostafa, A. M. A. *et al.* PbO–Sb_2O_3–B_2O_3–CuO glassy system: Evaluation of optical, gamma and neutron shielding properties. *Mater. Chem. Phys.* **258**, 123937 (2021).

23 Susoy, G. *et al.* The impact of Cr_2O_3 additive on nuclear radiation shielding properties of LiF–SrO–B_2O_3 glass system. *Mater. Chem. Phys.* **242**, 122481 (2020).

24 Al-Buriahi, M. S. *et al.* Structure, optical, gamma-ray and neutron shielding properties of NiO doped B_2O_3–$BaCO_3$–Li_2O_3 glass systems. *Ceram. Int.* **46**, 1711-1721 (2020).

25 Wu, Y., Hu, J., Feng, C., Chen, G. & Yang, J. Carborane-containing copolyester fibers with unique neutron shielding properties. *Mater. Des.* **172**, 107772 (2019).
